# Zusammenhang zwischen Substanzkonsum und Zwangsmaßnahmen auf psychiatrischen Stationen

**DOI:** 10.1007/s00115-021-01181-2

**Published:** 2021-09-07

**Authors:** Felix Betzler, Ariadne Brandt, Andreas Heinz, Henrik Walter

**Affiliations:** Klinik für Psychiatrie und Psychotherapie, Charité – Universitätsmedizin Berlin, corporate member of Freie Universität Berlin and Humboldt-Universität zu Berlin, Berlin, Deutschland

**Keywords:** Fixierung, Intoxikation, Alkohol, Cannabis, Drogen, Mechanical restraints, Intoxication, Alcohol, Cannabis, Drugs

## Abstract

**Ziel der Studie:**

Im Zuge der Minimierungsabsichten von Zwangsmaßnahmen ist ein gutes Verständnis für deren Einflussfaktoren elementar. Die vorliegende Arbeit untersucht den Zusammenhang zwischen Substanzkonsum und der Anwendung von Zwangsmaßnahmen.

**Methoden:**

Alle im Jahr 2019 durchgeführten Zwangsmaßnahmen der akutpsychiatrischen Stationen der Charité Campus Mitte wurden untersucht, mit Fokus auf den Zusammenhang mit Substanzgebrauchsstörungen.

**Ergebnisse:**

Bei 106 Fällen (92 Patienten) von insgesamt 1232 Behandlungsfällen (1131 Patienten) wurden Zwangsmaßnahmen angewendet, vorwiegend Unterbringung nach PsychKG (94) und nach BGB (21), seltener Isolation (23) oder Fixierung (18). Bei einem Drittel der Zwangsbehandlungsfälle lag eine akute Intoxikation, bei zwei Dritteln eine Substanzgebrauchsstörung in der Vorgeschichte vor, in beiden Fällen am häufigsten von Alkohol und/oder Cannabis. In der Gesamtzahl aller Behandlungsfälle hingegen (1232) lag die Anzahl von Intoxikationen bei 9 % und allgemein von Substanzgebrauchsstörungen bei 36 %.

**Schlussfolgerung:**

Die vorliegende Arbeit belegt die klinisch bekannte Assoziation zwischen Intoxikationen und dem Einsatz von Zwangsmaßnahmen.

## Abstract (en)

**Objective:**

The intention to minimize coercive measures requires a comprehensive understanding of the influencing factors. The aim of the present work was to examine the association between substance use and the use of coercive measures.

**Methods:**

All coercive measures carried out on acute psychiatric wards of the Charité Campus Mitte in 2019 were examined, with a particular focus on the association with substance use disorders.

**Results:**

In 106 cases (92 patients) out of a total of 1232 cases (1131 patients), coercive measures were used, mainly admissions according to PsychKG (94) and BGB (21), more rarely isolation (23) or mechanical restraint (18). Acute intoxication was present in approximately one third of cases with coercive measures and a history of substance use disorder in two thirds, most often with alcohol and/or cannabis. In contrast, 9% of 1232 treatment cases presented with acute intoxication and 36% with substance use disorders in general.

**Conclusion:**

The present work delivers empirical data confirming the clinically known association between intoxication and the use of coercive measures.

## Hintergrund

Während eines Aufenthaltes in stationären Psychiatrien werden in Deutschland bei etwa 8 % der Patienten eine oder mehrere Zwangsmaßnahmen angewendet. Mit 28 % ist ein relevanter Teil dieser Patienten wegen einer Suchterkrankung in Behandlung – und somit die zweithäufigste Gruppe, direkt nach Patienten mit einer Störung aus dem schizophrenen Formenkreis (etwa 31 % der Fälle). Dabei gehören die (körperliche) Fixierung und die räumliche Einschränkung der Bewegungsfreiheit bei Unterbringung innerhalb einer geschlossenen Station zu den am häufigsten angewendeten Zwangsmaßnahmen. Dies ergab sich in einer aktuellen Studie der Deutschen Gesellschaft für Psychiatrie und Psychotherapie, Psychosomatik und Nervenheilkunde (DGPPN), in der alle Zwangsmaßnahmen innerhalb 8 psychiatrischer Kliniken in Deutschland während einer 3‑monatigen Zeitspanne dokumentiert wurden [1]. Unklar hingegen bleibt trotz dieser Zahl, ob Suchterkrankungen mit Zwangsmaßnahmen in Verbindung stehen, da diese Diagnosen in vielen psychiatrischen Versorgungskliniken generell zu den häufigsten Diagnosen zählen.

Zwangsmaßnahmen werden in Deutschland gesetzlich reglementiert. Zuletzt sorgte das wegweisende Urteil des Bundesverfassungsgerichtes für die verpflichtende Einhaltung engmaschigerer juristischer Beurteilung und Dokumentation bei Zwangsmaßnahmen und somit für die Stärkung der Menschenrechte psychiatrischer Patienten mit einer bundesweit einheitlichen Regelung [4].

Zwangsmaßnahmen können für Betroffene eine psychisch und somatisch belastende und leider oft auch traumatisierende Erfahrung darstellen [15, 20]. Gleichzeitig können Sie bei strenger Indikationsstellung das letzte und einzige Mittel sein, Patienten, Mitpatienten und auch Mitarbeiter in psychiatrischen Kliniken vor körperlichen oder psychischen Schäden zu bewahren. Zahlreiche Arbeiten der letzten Jahre beschäftigen sich mit den Auswirkungen und Möglichkeiten einer Reduktion von Zwangsmaßnahmen, welche das Bestreben einer modernen psychiatrischen Praxis widerspiegeln, Zwangsmaßnahmen auf das absolut notwendige Minimum zu beschränken [14]. An der Berliner Charité, in Kooperation mit anderen Kliniken, wurden mehrere Interventionen zu der Reduktion, dem subjektiven Erleben und dem Verarbeiten von Zwangsmaßnahmen untersucht [6, 7, 18–24, 27, 30].

Eine Besonderheit der Berliner Allgemeinbevölkerung, welche sie von Kleinstädten, aber teilweise auch anderen deutschen Großstädten unterscheidet, ist unter anderem eine deutliche höhere Rate an Substanzkonsum im Vergleich zum bundesdeutschen Durchschnitt [16]. Zudem existiert in Berlin eine international bekannte und ausgeprägte Partyszene und Berlin wird deshalb in den Medien nicht selten als „Partyhauptstadt Europas“ bezeichnet [11]. Auch der Substanzkonsum innerhalb der Partyszene liegt deutlich über dem anderer deutscher Städte und zeigt auch im internationalen Vergleich mit anderen Großstädten hohe Prävalenzen [2, 26]. Der Zusammenhang von Alkoholkonsum und anderen Substanzgruppen mit aggressivem Verhalten (Selbst- und Fremdaggression) ist bekannt und gut beschrieben [12]. Zudem gibt es einen Zusammenhang zwischen Substanzkonsum und akuten psychotischen Zustandsbildern, die wiederum mit aggressivem Verhalten in Verbindung stehen können [9]. Entsprechend kann vermutet werden, dass sich die höhere Rate an Alkoholkonsum und anderer Substanzen in den Zahlen der durchgeführten Zwangsmaßnahmen in einem Krankenhaus der Maximalversorgung mit Einzugsgebiet im Zentrum der Stadt widerspiegelt.

Vor dem Hintergrund der Minimierungsabsichten von Zwangsmaßnahmen ist somit ein besseres Verständnis des Einflusses akuter Intoxikationen einerseits sowie vorbestehender Substanzgebrauchsstörungen andererseits notwendig. Die vorliegende Auswertung untersucht die in unserem Hause durchgeführten Zwangsmaßnahmen im Hinblick auf akute Intoxikationen sowie dem Vorliegen von Abhängigkeitserkrankungen oder Substanzmissbrauch in der Vorgeschichte der betroffenen Patienten. Hierfür wurden Zwangsmaßnahmen aus dem Jahr 2019, die auf den Akutstationen im Campus Mitte der Charité Universitätsmedizin Berlin durchgeführt wurden, systematisch ausgewertet.

## Methodik

Die für diesen Bericht erhobenen Daten stammen aus der schriftlichen Dokumentation (Dokumentationsbögen für Zwangsmaßnahmen, in denen u. a. Art, Grund und Dauer der Maßnahme dokumentiert werden sowie Entlassberichte und zusätzliche Dokumente wie z. B. Rettungsstellenscheine, Verlaufsberichte oder sonstige Dokumentationen durch Personal) aller Maßnahmen der Akutstationen der psychiatrischen Abteilung der Charité Campus Mitte aus dem Jahr 2019. Hierbei wurden a) Unterbringung nach BGB (§ 1906 Abs. 1, Bundesrecht), b) Unterbringung nach PsychKG (Berliner Landesrecht), c) Stationsgebot (d. h. Aufenthalt nur auf Station, auch Ausgang in Garten untersagt), d) räumliche Isolation, e) Fixierung, f) Zwangsmedikation und g) sonstige Zwangsmaßnahmen unterschieden. Zusätzliche Patientendaten und Informationen zur Begründung der Maßnahmen, zu aktuellen und früheren Diagnosen, zum Behandlungsverlauf und der Liegedauer sowie zum Substanzkonsum wurden aus den im Krankenhausdokumentationssystem hinterlegten digitalen Patientenakten und ärztlichen Entlassbriefen ergänzt. Dabei wurden auch Patienten berücksichtigt, bei denen eine oder mehrere Zwangsmaßnahmen bei mehreren stationären Aufenthalten durchgeführt wurden. Daher erscheinen in den folgenden Analysen mehr Fälle als Patienten.

## Ergebnisse

### Stichprobe

Wir analysierten alle Fälle, in denen die Informationen für unsere Fragestellungen (u. a. Daten zum Substanzkonsum) komplett vorlagen (85 % aller Fälle mit Zwangsmaßnahmen). Entsprechend konnten bei 106 Fällen (entspricht 92 Patienten) von insgesamt 1232 Behandlungsfällen (1131 Patienten) im Jahr 2019 eine oder mehrere Zwangsmaßnahmen ermittelt werden. Im Schnitt hatten die Patienten ein Alter von 42,4 Jahren, wobei der jüngste Patient 18 und der älteste Patient 93 Jahre alt waren. Dabei war die Geschlechterverteilung mit 45 (48,9 %) weiblichen Patientinnen relativ gleich. Im jeweiligen Fall betrug die durchschnittliche Liege- und stationäre Behandlungsdauer 36,2 Tage (*SD* = 55,5). Im Mittel wurde(n) die Zwangsmaßnahme(n) nach 5,3 Tagen (*SD* = 13,0) nach Aufnahme eingeleitet – wobei in 56,6 % der Fälle die Maßnahme(n) direkt am Aufnahmetag eingesetzt wurde und in weiteren 13,2 % der Fälle am darauffolgenden Tag.

**Figure 1:**
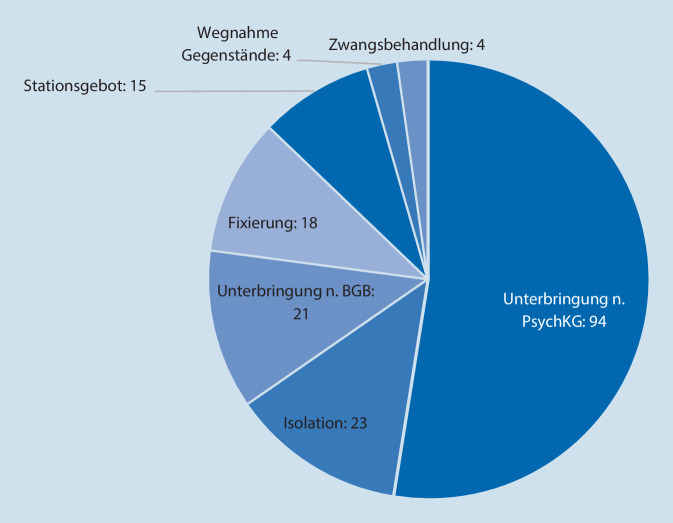

Meist wurden pro Fall eine (54,7 %) oder zwei (27,4 %) Zwangsmaßnahmen zur Behandlung eingesetzt. Das Maximum waren 6 Maßnahmen bei einem einzigen Fall. Am häufigsten wurde die Unterbringung nach PsychKG ausgesprochen, gefolgt von Isolation, der Unterbringung nach BGB, der körperlichen Fixierung und Stationsgebot. Nur vereinzelt erfolgte die Zwangsmedikation oder die Wegnahme (und Aufbewahrung) von Gegenständen, wie z. B. Feuerzeugen oder großer Bargeldsummen (Abb. 1). Zwangsmaßnahmen nach rechtfertigendem Notstand nach StGB kamen nicht zum Einsatz (ausgewertet wurden jedoch auch nur Fälle innerhalb psychiatrischer Stationen, bei denen die Zwangsmaßnahmen über PsychKG und BGB abgedeckt waren, während auf somatischen Stationen oder in den Rettungsstellen häufiger Maßnahmen über den rechtfertigenden Notstand erfolgen). In etwa 53,8 % der Fälle wurde der Patient durch die Polizei und/oder Feuerwehr vorgestellt, meist im Zusammenhang mit eigen- oder fremdgefährdenden Fehlhandlungen außerhalb der Klinik.

### Übersicht der Diagnosen

Grundsätzlich gab ein Großteil (63 %) der Patienten an, eine Vorgeschichte mit Substanzkonsum, -missbrauch oder -abhängigkeit zu haben. Dabei wird deutlich, dass von allen dokumentierten Substanzgebrauchsstörungen vor allem eine akute Alkoholintoxikation oder ein Einfluss von Cannabinoiden am häufigsten mit einer der durchgeführten Zwangsmaßnahmen zusammenfiel.

Alle Patienten erhielten mindestens eine, meist jedoch mehrere Diagnosen aus dem Bereich der psychischen Störungen. Es ist zu beachten, dass aufgrund hoher Komorbiditätsraten von weitaus mehr Diagnosen als Fällen berichtet wird.

**Figure 2:**
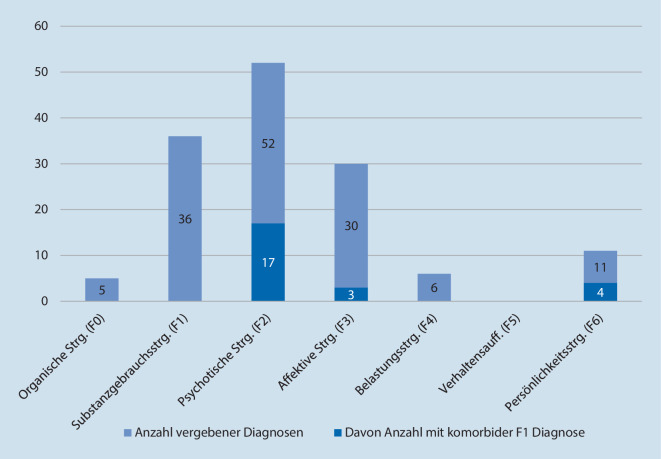

Am häufigsten vertreten bei der Durchführung einer oder mehrerer Zwangsmaßnahmen waren Diagnosen des schizophrenen Formenkreises (ICD-10 : F2, *n* = 52 Diagnosen), gefolgt von Störungen aus dem Kreis der Substanzgebrauchsstörungen (ICD-10 : F1, *n* = 36 Diagnosen). Diagnosen aus dem Bereich der organischen Störungen (ICD-10 : F0, *n* = 5 Diagnosen) waren selten, Diagnosen aus dem Bereich Verhaltensauffälligkeiten mit körperlichen Störungen und Faktoren gab es keine (ICD-10: F5). Erwartungsgemäß zeigten sich hohe Komorbiditätsraten, am häufigsten war die Kombination einer F2-Diagnose mit einer F1-Diagnose (32,7 % der Patienten). Entsprechend fand sich in den ärztlichen Dokumentationen der jeweiligen Fälle häufig eine substanzinduzierte psychotische Episode oder auch Erstmanifestationen einer manischen Episode nach Drogenkonsum. Die Verteilung der Störungsgruppen ist in Abb. 2 dargestellt.

### Muster des Substanzkonsums

**Figure 3:**
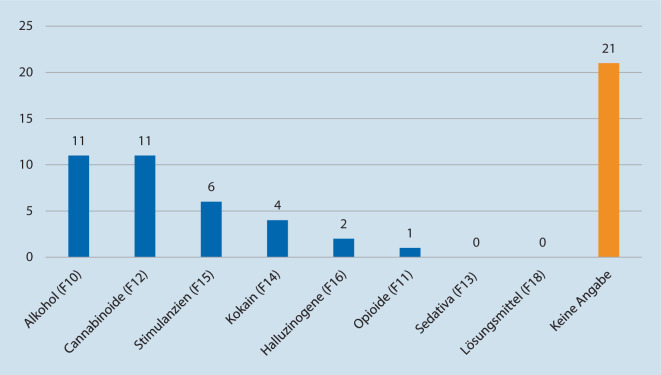

Bei 30 der insgesamt 106 Fälle (28,3 %) lag eine akute Substanzintoxikation vor, welche entweder durch Aussagen des jeweiligen Patienten bestätigt und/oder durch Alkoholatemtests oder Urinproben verifiziert werden konnte (Abb. 3). Dabei wird deutlich, dass Alkohol- und Cannabiskonsum bei weitem am häufigsten vorlagen (jeweils 34,4 % entsprechend je 11 Fällen). Die Einnahme von Stimulanzien wie (Meth‑)Amphetamin war am zweithäufigsten (18,8 % = 6 Fälle). Gar nicht vertreten waren die akute Einnahme von Sedativa oder Hypnotika und die Einnahme flüchtiger Lösungsmittel z. B. GHB oder GBL. Hierbei ist jedoch zu beachten, dass ein Nachweis dieser Substanzen innerhalb eines Blut- oder Urinscreenings nur maximal 12 h nach der Einnahme möglich ist [13]. Dies könnte maßgeblich zum Fehlen der Substanzgruppe innerhalb dieser Statistik beitragen.

Kritisch zu betrachten sind die Fälle (*n* = 21, entsprechend 19,8 % aller Fälle), in denen eine Aussage des Patienten oder eine medizinische Untersuchung zum Konsum verweigert wurde, wir aber teilweise klinisch den Verdacht auf eine Substanzintoxikation hatten. Zum Teil war die Exploration durch den Zustand des Patienten (fehlende Kooperation, erhöhte Agitiertheit und Aggression etc.) nicht möglich. Diese Fälle, zu denen keine Daten über aktuellen Konsum vorlagen oder erhoben werden konnten, enthalten mit hoher Wahrscheinlichkeit weitere Fälle von Substanzintoxikation, sodass die Häufigkeitsangaben in dieser Arbeit als konservativ (zu niedrig) einzuschätzen sind.

**Figure 4:**
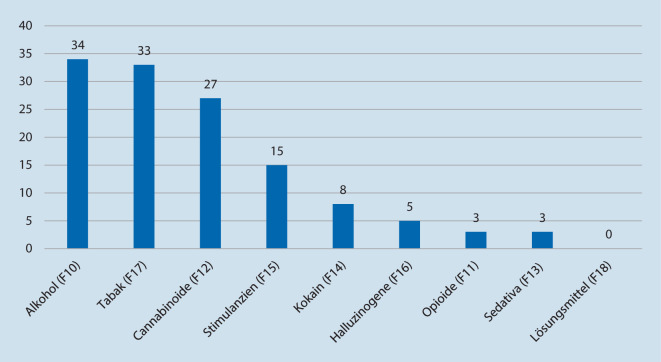

Bei vielen Patienten lag darüber hinaus Alkohol- und/oder Substanzkonsum in der Vergangenheit vor (Abb. 4). So gab es nur 39 Fälle, in denen kein Konsum in der Vorgeschichte dokumentiert wurde, 67 Fälle berichteten von regelmäßigem Substanzkonsum (Substanzabusus oder -abhängigkeit) in der Vorgeschichte. Etwa die Hälfte davon gab an, regelmäßig Alkohol konsumiert zu haben (34 Fälle) und etwas über ein Drittel konsumierte regelmäßig Cannabis (27 Fälle). Hinsichtlich anderer Substanzen gaben fast 3‑mal so viele Patienten eine Vorgeschichte mit regelmäßigem Stimulanzienkonsum an (15 Fälle), wie die, die akut bei Behandlung damit intoxikiert waren. Viele der Patienten hatten während früherer Behandlungen an der Charité aufgrund ihres Konsums Diagnosen einer Substanzgebrauchsstörung erhalten – unter anderem auch Diagnosen des multiplen Substanzgebrauch (F19). Bei 30 der 67 Fälle (44,8 %) mit positiver Substanzvorgeschichte wurde mehr als eine Substanz nachgewiesen oder benannt. Zwar sind die Angaben zu Mengen und Häufigkeit des vergangenen Konsums generell zu grob oder lückenhaft dokumentiert, um damit weitere statistische Auswertungen durchzuführen, allerdings lässt sich anhand der ärztlichen Dokumentationen ableiten, dass der Gebrauch einer oder mehrerer Substanzen häufig mit psychotischen Symptombildern assoziiert war.

### Bezug zur Gesamtzahl der Behandlungsfälle

Was die Gesamtzahl der Behandlungsfälle klinikweit betrifft, zeigt sich ein deutlich kleinerer Anteil akuter Intoxikationen (112 von insgesamt 1232 Fällen, entsprechend 9 % – wobei hier die o. g. Zahlen der Fälle mit Zwangsmaßnahme als Teilgröße enthalten sind).

Die Häufigkeit der F1-Diagnosen hingegen liegt mit 438 Fällen, in denen eine Substanzgebrauchsstörung als Haupt- und/oder Nebendiagnose kodiert wurde, klinikweit bei ca. einem Drittel (36 %).

## Diskussion

Zwangsmaßnahmen in der Psychiatrie gelten sowohl in der Praxis als auch in der Forschung als persönlich sehr belastende Behandlungsmethode der letzten Wahl [15, 20]. In der Literatur besteht Einigkeit darüber, dass eine hohe Aggressionsbereitschaft sowie Diagnosen aus dem Bereich der organischen Störungen und/oder des schizophrenen Formenkreises mit der Durchführung von Zwangsmaßnahmen statistisch signifikant assoziiert sind [1, 10, 25]. Hinsichtlich des Zusammenhangs mit der Diagnose einer Substanzgebrauchsstörung findet sich eine heterogene Datenlage, in der Mehrheit wird jedoch kein Zusammenhang beschrieben. Während Walker und Kollegen (2019) in einer Metaanalyse unter Erwachsenen mit F1-Diagnose insgesamt sogar ein reduziertes Risiko für Zwangsmaßnahmen beschreiben [29], liegt bei ihrer Analyse von Jugendlichen mit F1-Diagnose hingegen ein erhöhtes Risiko vor [28]. Eine andere Arbeit aus der Schweiz differenziert nach Art der Substanz und findet bei Alkoholabhängigkeit (bei Frauen) ein erhöhtes, bei sonstigen Substanzen hingegen ein erniedrigtes Risiko [5].

Im Rahmen der psychiatrischen Stationen der Charité Berlin Campus Mitte war der Einsatz einer oder mehrerer Zwangsmaßnahmen bei Patienten mit einer Diagnose aus dem Bereich der schizophrenen und sonstigen psychotischen Störungen (F2) mit 52 Fällen (49 %) am häufigsten vertreten. Die zweithäufigste Diagnosegruppe war die Gruppe der Substanzgebrauchsstörungen (F1) mit 36 Fällen (34 %) – oft in Form einer Komorbidität. Der Anteil an Patienten mit F1-Diagnose fällt im Vergleich zu anderen Studien aus dem europäischen Raum relativ hoch aus: So wurden in einer groß angelegten Studie im Zentrum für Psychiatrie in Südwürttemberg, einer im ländlichen Bereich gelegenen Einrichtung, alle Zwangsmaßnahmen des Jahres 2007 dokumentiert. Aus den insgesamt 576 Fällen, in denen eine Zwangsmaßnahme eingesetzt wurde, hatten dort nur 2,6 % eine Substanzgebrauchsstörung [10]. Im Bericht von Adorjan und Kollegen, in dem deutschlandweit Daten von 8 Kliniken ausgewertet wurden, darunter auch Universitätskliniken [1], wiesen wiederum prozentual 28 % der betroffenen Patienten eine Substanzgebrauchsstörung auf.

Die Prävalenzen variieren also deutlich – hierzu sind unterschiedliche Gründe denkbar. Zum einen ist der Substanzkonsum in Berlin höher als im bundesdeutschen Durchschnitt und Berlin gilt als Metropole, die für ihre Feierkultur und entsprechenden Substanzgebrauch bekannt ist. Innerhalb der Großstadt ist eine erhöhte Präsenz und Verfügbarkeit von Substanzen sowie die Entwicklung von Subkulturen und Drogenszenen im Vergleich zu ländlicheren Gegenden beschrieben [2, 16], was sich aus o. g. Gründen vermutlich auch in den Zwangsmaßnahmen und deren Ursachen widerspiegelt. Während die akute Intoxikation von Alkohol sowie Cannabis in unserer Stichprobe besonders relevant war, hatte auch ein Großteil der Patienten in ihrer Vorgeschichte Substanzen eingenommen (darunter Kokain, Halluzinogene und Stimulanzien). Dabei lagen die jeweiligen Prävalenzen pro Substanzgruppe weit über dem Durchschnitt innerhalb der deutschen Population: In einer epidemiologischen Studie gaben z. B. 0,6 % der deutschen Allgemeinbevölkerung an, in den letzten 12 Monaten Kokain eingenommen zu haben. Die Lebenszeitprävalenz der repräsentativen Stichprobe lag bei 2,5 % [17]. Innerhalb unserer vorliegenden psychiatrischen (selektiven) Stichprobe gaben demgegenüber 7,5 % der Fälle an, Kokain konsumiert zu haben oder erhielten im Vorfeld eine Diagnose aus dem Bereich (F14.xx). Hingegen wurde nur bei 1 % der Gesamtzahl der Behandlungsfälle eine solche Diagnose dokumentiert. Diese Gegenüberstellung kann jedoch nur als Orientierung dienen, da die Zwangsbehandlungsfälle umfassender analysiert wurden (händische Durchsicht der Verlaufsdokumentationen, Arztbriefe, sonstiger Dokumentationen) als durch die maschinelle Auswertung der Behandlungsdaten für die Gesamtzahl der Fälle, unter denen sich ggf. Fälle befinden, bei denen z. B. ein probatorischer Kokainkonsum in der Vorgeschichte angegeben, jedoch nicht als *Diagnose* erfasst wurde (Prävalenz vs. Diagnose).

Überdies werden die oben berichteten Prävalenzschwankungen der Zwangsmaßnahmen auch durch die klinische Schwerpunktsetzung der jeweiligen Krankenhäuser sowie durch das Dokumentationsverhalten der Mitarbeiter beeinflusst.

Es muss bei der Diagnosenverteilung unter Zwangsmaßnahmefällen jedoch auf die limitierte Aussagekraft hingewiesen werden, da der Anteil von F1-Diagnosen in der Gesamtzahl der Behandlungsfälle in den meisten psychiatrischen Versorgungskliniken zu den häufigsten Diagnosen zählt. Dies ist auch in unserer Klinik der Fall (zweithäufigste Diagnose nach F3-Diagnosen) und die Zahl der F1 als Haupt- und/oder Nebendiagnose ist an sich häufig zu finden (36 % aller Behandlungsfälle). Noch aussagekräftiger scheint das gehäufte Auftreten akuter Intoxikationen im Zusammenhang mit Zwangsbehandlungen zu sein, welches unter den Zwangsbehandlungsfällen deutlich höher liegt als in der Gesamtzahl der klinikweiten Behandlungsfälle (28 % vs. 9 %).

Bemerkenswert ist hierbei der hohe Anteil an Cannabisintoxikationen unter den von Zwangsmaßnahmen betroffenen Patienten, da Cannabiskonsum unter psychisch gesunden Probanden eher zu einer Verringerung der Aggressivität führt, im Gegensatz zu Alkoholkonsum, bei dem eine erhöhte Aggressivität zu beobachten ist [8]. Denkbar ist bei den vorliegenden Ergebnissen, dass der hohe Anteil von Patienten mit Diagnosen aus dem schizophrenen Formenkreis die prominente Rolle von Cannabis in der Gruppe der substanzkonsumassoziierten Zwangsmaßnahmen erklärt, da bei dieser Personengruppe ein Cannabiskonsum zu einer Verstärkung der psychotischen Symptomatik führen kann [9], was wiederum in erhöhter Aggressivität und dadurch bedingter Notwendigkeit von Zwangsmaßnahmen münden könnte. Inwieweit synthetische Cannabinoide, die oftmals viel potenter sind als herkömmliche Cannabisprodukte, diesbezüglich eine Rolle spielen, lässt sich durch die vorliegenden Daten nicht beantworten, da die Erfassung solcher Substanzen zu unscharf ist: Diese Substanzen obliegen den Angaben in der Anamnese oder sonstigen Hinweisen (Fremdanamnese, Identifizierung durch beschriftete Tütchen etc.), da sie nur in seltenen Fällen durch herkömmliche Screeninginstrumente detektiert werden [3]. Des Weiteren kann ein Teil der Zwangsmaßnahmen (insbesondere die, die nicht am Aufnahmetag stattfanden, sondern in den Folgetagen) möglicherweise auch durch den *Wegfall* des Substanzkonsums erklärt werden im Sinne von Craving und damit verbundener Steigerung der Aggressivität.

Ein limitierender Aspekt der vorliegenden Arbeit ist, dass lediglich eine Assoziation von Substanzkonsum und Zwangsmaßnahmen dargestellt ist – ein kausaler Zusammenhang ist damit nicht direkt nachweisbar. Zudem ist die Stichprobengröße zwar ausreichend für eine veranschaulichende deskriptive Darstellung der Ergebnisse, nicht aber für eine Analyse der Daten anhand statistischer Verfahren. Da es sich um eine retrospektive Auswertung handelt, konnten nur die ohnehin dokumentierten Basisparameter berücksichtigt werden. Zukünftige Studien mit prospektivem Design und größerer Stichprobe könnten hierzu verlässlichere Aussagen treffen. Zudem ist die Aussagekraft eines Hauptparameters der vorliegenden Arbeit (vorliegen einer F1-Diagnose) aus oben beschriebenen Gründen eingeschränkt. Bei dem Vergleich zwischen Zwangsbehandlungsfällen und der Gesamtzahl der Behandlungsfälle ist zu beachten, dass die Zwangsbehandlungsfälle eine Teilgröße der Gesamtzahl der Fälle sind. Letztlich untersucht die vorliegende Arbeit auch nur die Fälle einer einzelnen Klinik. Regionale Unterschiede und eine unterschiedliche thematische Ausrichtung bleiben hierdurch unberücksichtigt.

Zusammenfassend haben wir Hinweise auf einen Zusammenhang von Zwangsmaßnahmen mit Substanzkonsum, insbesondere mit akuten Intoxikationen gefunden, wobei Alkohol und Cannabis dominieren. Allerdings muss diese prominente Rolle ins Verhältnis zu den ohnehin hohen Prävalenzen dieser beiden Substanzen in der Allgemeinbevölkerung gesetzt werden. In Relation zur Prävalenz kommt daher auch den Stimulanzien/Kokain proportional eine hohe Bedeutung zu. Die Ergebnisse legen zudem nahe, dass die Minimierungsabsichten von Zwangsmaßnahmen selbst bei voller Ausschöpfung vermutlich nicht ausreichen, um die intoxikationsbedingten Zwangsmaßnahmen gänzlich vermeiden zu können [6, 7, 18]. Indes ist bei dem hohen Anteil von Intoxikationen eine Erweiterung des Fokus zwangspräventiver Interventionen auf den Bereich Substanzkonsum sinnvoll und sollte Gegenstand weiterer Minimierungsbemühungen sein. Zudem sollte sich der Aspekt prominent in der Information und Psychoedukation der betroffenen Patientenklientel wiederfinden. Insbesondere bei psychiatrischen Patienten mit F2-Diagnose zeigen sich im klinischen Alltag häufig Bagatellisierungstendenzen gegenüber einem subjektiv „beruhigenden Effekt“ von Cannabis. Wenngleich dies bei gesunden Probanden tatsächlich beschrieben ist, weisen die vorliegenden Daten darauf hin, dass Cannabisintoxikationen bei Vorerkrankten bezüglich Zwangsmaßnahmen eine negative Rolle zukommen könnte.

## Fazit für die Praxis

Der Zusammenhang von Intoxikationen und Substanzgebrauchsstörungen mit dem Einsatz von Zwangsmaßnahmen verdeutlicht die Wichtigkeit dieses Aspekts in der Psychoedukation der entsprechenden Patientenklientel – insbesondere im Hinblick auf den hohen Anteil der Cannabisintoxikationen unter den oft komorbid psychotisch betroffenen Fällen und der häufig beobachteten Bagatellisierungstendenz unter psychisch erkrankten Patienten.
